# Enfortumab Vedotin plus Pembrolizumab as First-line Treatment in Recurrent or Metastatic Head and Neck Squamous Cell Carcinoma: Results from EV-202 Cohort 9

**DOI:** 10.1158/1078-0432.CCR-25-4650

**Published:** 2026-05-05

**Authors:** Paul L. Swiecicki, Glenn J. Hanna, Jessica L. Geiger, Takao Fujisawa, Missak Haigentz, Yoshitaka Honma, Justine Y. Bruce, Kaoru Tanaka, Kei Muro, Priyanka Bhateja, Jason Kaplan, Shubin Liu, Seema Gorla, Michele Wozniak, Ryan Dillon, Salaheldin Hamed, Changting Meng, Marya F. Chaney, Ari J. Rosenberg

**Affiliations:** 1 Division of Hematology/Oncology, Department of Internal Medicine, University of Michigan Rogel Cancer Center, Ann Arbor, Michigan.; 2 Department of Medical Oncology, Dana-Farber Cancer Institute, Boston, Massachusetts.; 3 Department of Hematology and Medical Oncology, Taussig Cancer Center, Cleveland Clinic, Cleveland, Ohio.; 4 Head and Neck Medical Oncology, National Cancer Center Hospital East, Kashiwa, Japan.; 5 Rutgers Cancer Institute, Robert Wood Johnson Medical School, Rutgers University, New Brunswick, New Jersey.; 6 Department of Head and Neck, Esophageal Medical Oncology, National Cancer Center Hospital, Tokyo, Japan.; 7 Division of Hematology, Medical Oncology, and Palliative Care, Department of Medicine, University of Wisconsin–Madison, Madison, Wisconsin.; 8 Department of Medical Oncology, Kindai University, Osaka, Japan.; 9 Department of Clinical Oncology, Aichi Cancer Center Hospital, Nagoya, Japan.; 10 Department of Medical Oncology, The Ohio State University Wexner Medical Center, Columbus, Ohio.; 11 Astellas Pharma, Inc., Northbrook, Illinois.; 12 Pfizer Inc., Bothell, Washington.; 13 Merck & Co., Inc., Rahway, New Jersey.; 14 Department of Medicine, Section of Hematology and Oncology, University of Chicago, Chicago, Illinois.

## Abstract

**Purpose:**

To assess the safety and efficacy of enfortumab vedotin (EV) and pembrolizumab as first-line treatment for recurrent or metastatic head and neck squamous cell carcinoma (R/M HNSCC).

**Patients and Methods:**

In this open-label, multicohort, phase II study (NCT04225117), the R/M HNSCC cohort (cohort 9) received EV (1.25 mg/kg IV) on days 1 and 8 and pembrolizumab (200 mg IV) on day 1 in 21-day cycles. Eligible patients had R/M HNSCC, programmed cell death ligand 1 (PD-L1) combined positive score (CPS) ≥1, and no prior systemic therapy in the R/M setting. The primary endpoint was the investigator-assessed confirmed objective response rate (cORR) per Response Evaluation Criteria in Solid Tumors version 1.1. Secondary endpoints included duration of response (DOR), progression-free survival (PFS), overall survival (OS), and safety.

**Results:**

The primary analysis included 41 patients. Of these, 39% had PD-L1 CPS 1–19, and 61% had CPS ≥20. The median interquartile range nectin-4 expression H score was 185 (110–255). In patients with oropharyngeal squamous cell carcinoma (*n* = 14), 78.6% had p16–positive disease. The median follow-up for OS was 11 months. Sixteen patients achieved a response, and cORR was 39% [95% confidence interval (CI), 24.2–55.5]. The complete response rate was 9.8%. Median DOR was not estimable, and the estimated 6-month DOR rate was 81.7% (95% CI, 42–95.4). The median PFS was 5.1 months (95% CI, 3.5–not estimable). Grade ≥3 treatment-related adverse events were reported in 41.5% of patients, most commonly maculopapular rash (7.3%).

**Conclusions:**

EV with pembrolizumab demonstrated promising clinical activity as first-line treatment in patients with PD-L1 CPS ≥1 R/M HNSCC. Safety results reinforced the manageable tolerability profile of EV with pembrolizumab.

**Translational Relevance**

Patients with recurrent or metastatic head and neck squamous cell carcinoma (R/M HNSCC) have limited first-line treatment options. The combination of enfortumab vedotin (EV), a nectin-4–directed antibody–drug conjugate, and pembrolizumab, a programmed cell death 1 (PD-1) inhibitor, demonstrated clinically meaningful efficacy in patients with untreated locally advanced or metastatic urothelial carcinoma, but its effect on other locally advanced or malignant solid tumors is unclear. This cohort of the EV-202 study demonstrated a promising confirmed objective response rate of 39%, with durable responses observed in some patients and a manageable safety profile in line with other studies of EV with pembrolizumab. Further evidence is required to determine if cotargeting nectin-4 and the PD-1/programmed cell death ligand 1 axis will be a viable strategy to treat R/M HNSCC and beyond this specific regimen.

## Introduction

Globally, head and neck squamous cell carcinoma (HNSCC) is the eighth most common cancer, accounting for an estimated 790,000 new cases per year (1). Approximately 25% to 50% of patients with HNSCC develop recurrent or metastatic (R/M) disease after primary treatment (2, 3). Based on version 1 of the 2026 NCCN Clinical Practice Guidelines in Oncology (NCCN Guidelines), the recommended first-line treatment option for R/M non-nasopharyngeal HNSCC is the programmed cell death 1 (PD-1) receptor inhibitor pembrolizumab in combination with platinum-based chemotherapy or pembrolizumab monotherapy in patients with a programmed cell death ligand 1 (PD-L1)–positive tumor defined as a combined positive score (CPS) ≥1 (4). In routine clinical practice, however, the choice between these recommended options for patients with PD-L1 CPS 1 to 19 disease varies considerably, reflecting differences in evidence interpretation, patient selection, and institutional treatment patterns.

Despite these treatment advances, the prognosis for patients with R/M HNSCC remains poor, with a median overall survival (OS) of 11.5 to 13 months and an objective response rate (ORR) of 17% to 36% in patients treated with pembrolizumab, with or without platinum-based chemotherapy (5, 6). The prognosis of patients with R/M HNSCC can also be affected by other factors, including human papillomavirus (HPV) positivity (7).

Nectin-4 is a cell-adhesion molecule that is highly expressed in many different tumor types, including HNSCCs (8, 9). The nectin-4–directed antibody–drug conjugate enfortumab vedotin (EV; ref. 9) is being investigated in the EV-202 study, an international phase II basket study evaluating the efficacy and safety of EV as monotherapy or in combination with pembrolizumab in locally advanced or malignant solid tumors, including breast cancer, lung cancer, gastric or esophageal cancer, and head and neck cancer (10). In cohort 5 of the EV-202 study, EV demonstrated clinically meaningful activity as monotherapy in patients with heavily pretreated unresectable R/M head and neck cancer (11).

Preclinical evidence suggests that EV can potentiate the effect of immune checkpoint inhibitors such as pembrolizumab through their separate and complementary engagement of the immune system (12, 13). EV in combination with pembrolizumab has previously demonstrated clinically meaningful efficacy in patients with untreated locally advanced or metastatic urothelial carcinoma (la/mUC) compared with chemotherapy and is the standard of care as first-line treatment for la/mUC (14).

Although the efficacy and safety of EV with pembrolizumab are well characterized in la/mUC, the effect of this combination in R/M HNSCC is unknown. Here, we present the primary results from cohort 9 of the EV-202 study, which evaluated first-line EV with pembrolizumab in PD-L1 CPS ≥1 R/M HNSCC.

## Patients and Methods

### Ethics

This study was approved by independent Institutional Review Boards and ethics committees and was conducted according to the International Council for Harmonisation guidelines for Good Clinical Practice and the Declaration of Helsinki. Patients provided written informed consent before enrolling in the trial.

### Study design

EV-202 (NCT04225117) is an open-label, multicenter, multicohort, phase II study with cohort 9 specifically assessing the antitumor activity and safety of EV in combination with pembrolizumab as first-line treatment in patients with PD-L1 CPS ≥1 R/M HNSCC (Supplementary Fig. S1). In this cohort, a two-stage Bayesian optimal design was used, wherein an interim analysis of antitumor activity was conducted when 20 patients treated with EV were evaluable for an investigator-assessed response. The minimum number of responders required to proceed to stage 2 was based on the reference confirmed ORR (cORR) of 20% based on historical data (5, 6) and the target cORR. Cohort 9 aimed to include 40 evaluable patients in the final analysis of the R/M HNSCC cohort, with a minimum of 14 responders to declare promising antitumor activity, based on historical controls (5, 6).

### Study intervention

Patients received intravenous EV 1.25 mg/kg on days 1 and 8 and pembrolizumab 200 mg on day 1 of each 21-day cycle until a study discontinuation criterion was met, including documented radiologic disease progression, the start of a new anticancer therapy, unacceptable toxicity, or a continuous dose interruption >6 months. Patients received up to 35 cycles of pembrolizumab. There was no upper limit on the number of EV administrations. Dose modifications (EV only) and interruptions for treatment-related adverse events (TRAEs) were permitted per protocol. Patients who experienced unacceptable EV- or pembrolizumab-related toxicity may have remained in the study on pembrolizumab or EV monotherapy, respectively.

### Patients

Patients considered adults as per local regulations with histologically confirmed R/M HNSCC were included. Patients must have had a PD-L1 CPS ≥1 as determined by local testing or central testing with the 22C3 pharmDx immunohistochemical assay if local testing was unavailable. The use of the 22C3 pharmDx assay was not required for local PD-L1 testing. In addition, patients must have had measurable disease by Response Evaluation Criteria in Solid Tumors version 1.1 (RECIST v1.1), an Eastern Cooperative Oncology Group (ECOG) performance status (PS) of 0 or 1, and received no prior systemic therapy in the R/M setting (except for systemic therapy completed >6 months prior if given as part of multimodal treatment for locally advanced disease). Patients with oropharyngeal cancers must have had results from p16 immunohistochemical testing. Exclusion criteria included preexisting sensory or motor neuropathy grade ≥2, uncontrolled diabetes mellitus, active central nervous system metastases, prior treatment with EV, a history of pneumonitis or interstitial lung disease, ongoing grade 3 or greater hypothyroidism, a history of an allogeneic tissue or solid organ transplant, and active autoimmune disease that had required systemic treatment in the past 2 years.

### Outcomes and endpoints

The primary endpoint was cORR per RECIST v1.1 as determined by the investigator. ORR was defined as the number of patients whose best objective response was a confirmed complete response (CR) or partial response (PR). The secondary endpoints included duration of response (DOR), disease control rate (DCR), and progression-free survival (PFS) per RECIST version 1.1 by investigator assessment, OS, and safety variables.

Exploratory outcomes included ORR, DOR, DCR, and PFS per blinded independent central review (BICR) and exploration of genomic and/or other biomarkers (such as nectin-4 expression as measured by H score) as correlates with treatment outcomes, immunogenicity, pharmacokinetics [EV and monomethyl auristatin E (MMAE)], and patient-reported outcomes (PROs).

### Assessments

DOR was defined as the time from the first documented response (CR or PR) to the date of the first documented disease progression or death. DCR was the proportion of patients whose best objective response was CR, PR, or stable disease. PFS was defined as the time from the start of the study treatment to progressive disease or death due to any cause, whichever occurred first. Adverse events (AEs) were monitored until 30 days after the last dose of study medication and were graded based on the NCI’s Common Terminology Criteria for Adverse Events version 4.03. Predefined TRAEs of special interest for EV were analyzed and reported as composite terms composed of standardized Medical Dictionary for Regulatory Activities version 27.1 queries that included a number of preferred AEs, as previously defined in clinical studies of EV (15). Treatment-emergent AEs of special interest for pembrolizumab were evaluated using previously described criteria for pembrolizumab monotherapy (16).

Nectin-4 expression was retrospectively assessed in fresh or archival formalin-fixed, paraffin-embedded pretreatment tumor samples using a validated immunohistochemical assay, as described previously (11). Nectin-4 expression was detected with the proprietary M22-321b41.1 antibody and assessed by H score. Assessment of the remaining exploratory outcome measures is reported in the Supplementary Methods.

### Statistical analyses

One planned interim analysis was conducted to evaluate cORR at the time when 20 patients had evaluable tumor response data. If at least five patients had responded, the study proceeded to a second stage in which enrollment continued until the planned size of the study cohort was reached. Based on the planned sample size of 40 patients for this study, 14 patients were the minimum number of responders required to claim promising antitumor activity. Survival endpoints and PROs were analyzed in all patients who received any study drugs. ORR, DOR, and DCR were analyzed in all dosed patients with measurable disease at baseline per investigator assessment who had more than two postbaseline response assessments or were no longer in follow-up for response at the time of analysis. Safety data were calculated from the safety analysis set, which included all patients who received at least one dose of EV and pembrolizumab. Data were summarized descriptively for continuous data and with frequencies/percentages for categorical data. Time-to-event endpoints and median survival were estimated using the Kaplan–Meier method with two-sided 95% confidence intervals (CIs). For ORR and DCR, two-sided 95% CIs were calculated using the Clopper–Pearson method. All data processing, summarization, and analyses were performed using SAS Version 9.4 or higher.

## Results

### Patient disposition

A total of 41 patients were enrolled and received at least one dose of EV and pembrolizumab and were included in the efficacy, safety, and pharmacokinetic analyses.

**Figure 1.**
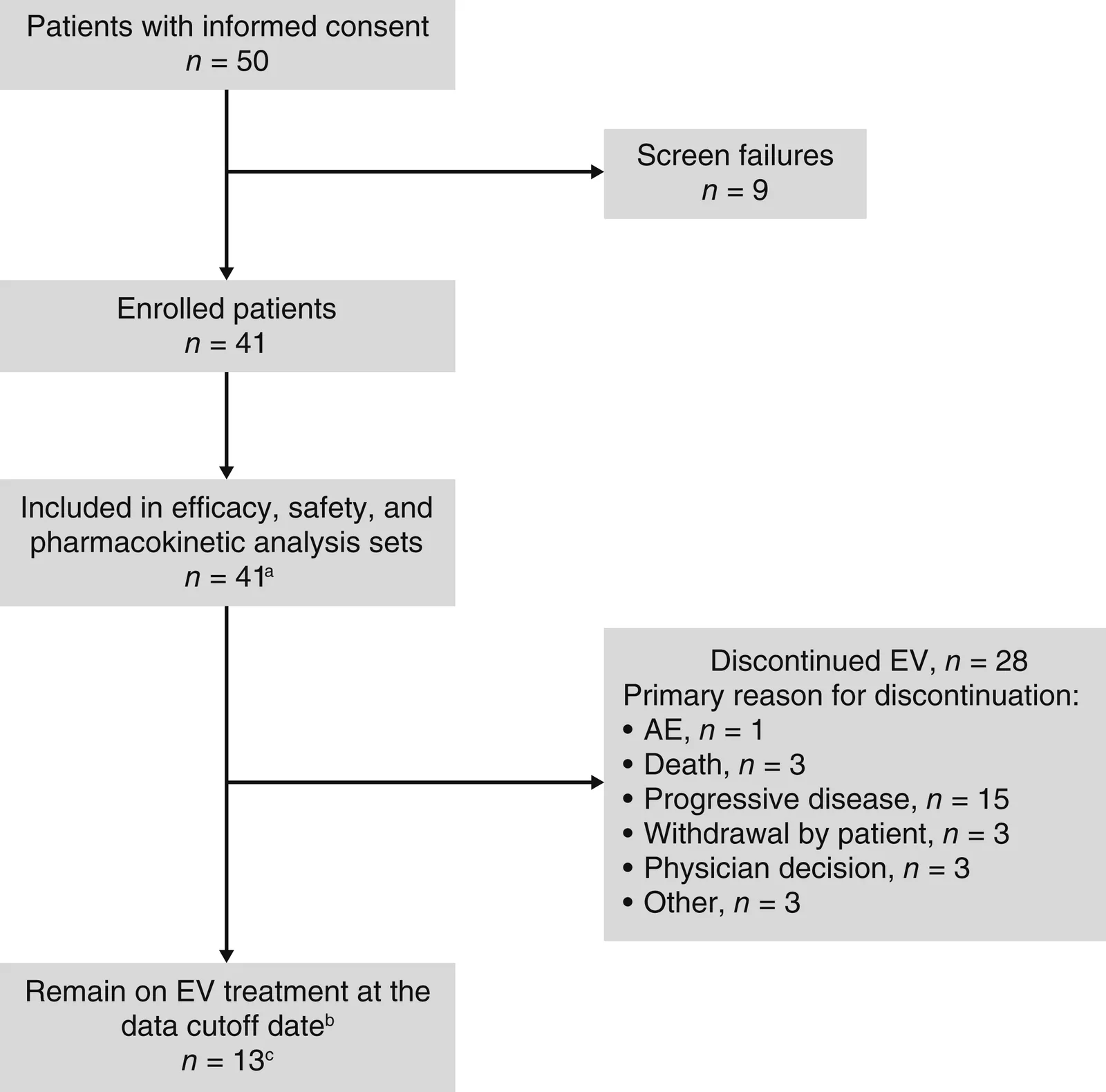
Patient disposition. ^a^Three patients were discontinued from treatment (all due to death) before the first postbaseline scan and did not have response data available. ^b^Data cutoff: February 25, 2025. ^c^Two patients discontinued pembrolizumab only due to AEs.

As of the data cutoff date (February 25, 2025), 13 patients remained on EV and 11 patients remained on pembrolizumab; 2 additional patients discontinued pembrolizumab due to AEs (Fig. 1). The most common (36.6%) reason for treatment discontinuation for both EV and pembrolizumab was progressive disease. The median (95% CI) follow-up period for OS was 11 (9.5–12.1) months.

### Patient characteristics

Most patients were male (75.6%), were White (70.7%), and had a median (range) age of 67 (51–82) years (Table 1). Nineteen patients (46.3%) had an ECOG PS score of 1. The tumor location was the oral cavity in 53.7% of patients. In the 14 patients with oropharyngeal squamous cell carcinoma, 11 (78.6%) had p16–positive disease. Most (61%) patients had PD-L1 CPS ≥20, and 39% had a CPS of 1 to 19. Nearly all patients with available data [35/36 (97.2%)] expressed nectin-4 in their submitted tumor tissue (nectin-4 H score >0). Nectin-4 was expressed at mid to high levels, with a median (interquartile range) nectin-4 H score of 185 (110–255). The representativeness of study participants to the overall population of patients with HNSCC is presented in Supplementary Table S1.

**Table 1.** Demographic and disease characteristics.

Characteristic, *n* (%)	R/M HNSCC (*n* = 41)
Male	31 (75.6%)
Race	​
White	29 (70.7)
Black or African American	2 (4.9)
Asian	9 (22)
Not reported	1 (2.4)
Median (range) age, years	67 (51–82)
Region	​
North America	32 (78)
Asia	9 (22)
ECOG PS	​
0	22 (53.7)
1	19 (46.3)
Albumin level	​
<LLN	6 (14.6)
≥LLN	33 (80.5)
Missing	2 (4.9)
Median (range) time since initial diagnosis, months^a^	9.3 (0.8–117.8)
Location	​
Oral cavity	22 (53.7)
Larynx	4 (9.8)
Oropharynx	14 (34.1)
Hypopharynx	1 (2.4)
Anatomic staging at screening	​
III	9 (22)
IV	10 (24.4)
IVA	6 (14.6)
IVB	5 (12.2)
IVC	8 (19.5)
Unknown	2 (4.9)
Other	1 (2.4)
p16 status^b^	​
Negative	29 (70.7)
Positive	11 (26.8)
Unknown	1 (2.4)
Nectin-4 IHC H score (tissue)^c^^,^^d^	​
H score >0	35 (97.2)
Median (IQR)	185 (110–255)
PD-L1 IHC CPS (tissue)^e^	​
1 to <20	16 (39)
≥20	25 (61)

Abbreviations: IHC, immunohistochemistry; IQR, interquartile range; LLN, lower limit of normal.

a Defined as the time from initial diagnosis to the date of the first dose of EV and pembrolizumab.

b For oropharyngeal squamous cell carcinoma only, patients without oropharyngeal cancer were considered HPV–negative.

c Assessed using a validated immunohistochemical assay (M22-321b41.1).

d Thirty-six patients with evaluable tumor tissue. H score range 0–300.

e Based on local laboratory PD-L1 test results.

### Treatment duration and exposure

The median (range) duration of overall treatment exposure was 4.9 (0.7–13.6) months for EV and 4.9 (0.7–15.4) months for pembrolizumab. The median (range) number of treatment cycles was 6 (1–17) for EV and 6 (1–21) for pembrolizumab. As 13 and 11 patients remained on EV and pembrolizumab treatment at the data cutoff date, respectively, the maximum treatment exposure and number of treatment cycles will increase with further follow-up. The median (range) relative dose intensity for EV was 89% (42.1–100.2). EV doses were reduced in 24.4% of patients, whereas 31.7% and 7.3% of patients had EV and pembrolizumab dose interruptions, respectively.

### Antitumor activity

**Figure 2.**
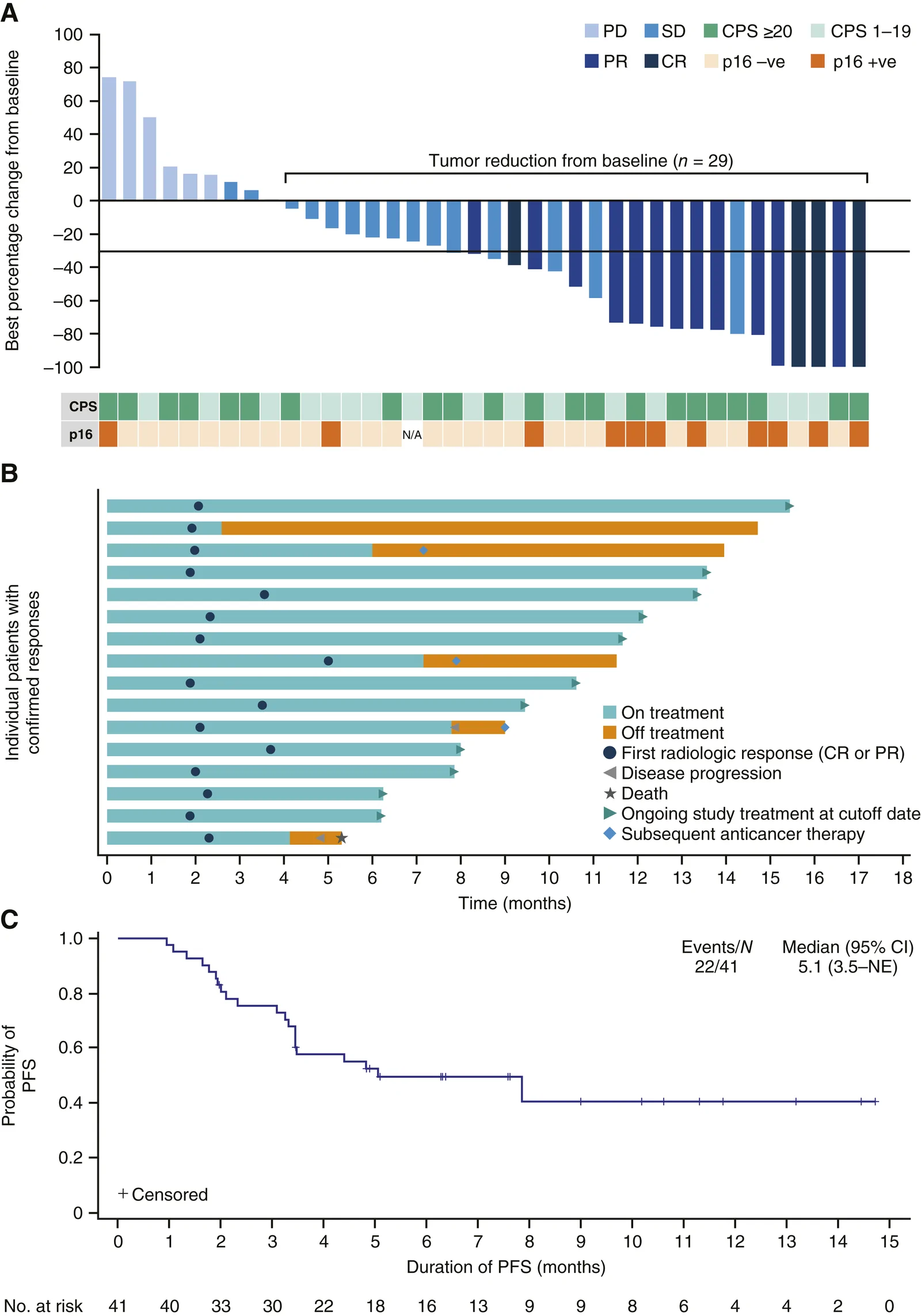
Efficacy per investigator assessment. **A,** Best change from baseline in the size of the target lesion. **B,** Time to response and DOR in patients with confirmed responses. **C,** Kaplan–Meier plots of PFS. N/A, not applicable; PD, progressive disease; SD, stable disease.

Postbaseline tumor assessments were conducted in 38 patients as 3 patients were discontinued from treatment before the first postbaseline scan. Sixteen patients achieved a response, and the cORR (95% CI) by investigator assessment was 39% (24.2–55.5; Table 2). The best overall response was CR for 4 patients (9.8%) and PR for 12 patients (29.3%). Tumor reductions from baseline were observed in 29 (76.3%) patients (Fig. 2A). Among patients with a confirmed PR or CR, the median (range) time to response was 2.3 (1.9–5) months (Fig. 2B). The median (95% CI) DOR was not estimable [5.8–not estimable (NE); Supplementary Fig. S2], and the 6-month DOR rate (95% CI) was 81.7% (42–95.4). The confirmed DCR (95% CI) was 75.6% (59.7–87.6).

**Table 2.** Summary of response by investigator assessment.

​	R/M HNSCC (*n* = 41)
Best confirmed overall response, *n* (%)^a^	​
Confirmed CR	4 (9.8)
Confirmed PR	12 (29.3)
Stable disease	15 (36.6)
Progressive disease	7 (17.1)
Not evaluable	3 (7.3)
cORR, *n* (%)^b^	16 (39)
95% CI^c^	24.2–55.5
Disease control rate, *n* (%)^d^	31 (75.6)
95% CI^c^	59.7–87.6

a Definition of best overall response followed RECIST v1.1. Complete and PRs must have been confirmed by two scans, a minimum of 4 weeks apart. The minimum duration for stable disease was 49 days after the date of the first dose of EV or pembrolizumab.

b cORR was defined as the proportion of patients whose best overall response was a confirmed CR or a PR as per RECIST v1.1.

c Calculated using the Clopper–Pearson method.

d Disease control rate was defined as the proportion of patients who have a best overall response of confirmed CR, PR, or stable disease (≥7 weeks).

The median (95% CI) PFS by investigator assessment was 5.1 (3.5–NE) months with a 6-month PFS rate (95% CI) of 49.5% (33.2–63.9; Fig. 2C). The median (95% CI) OS was NE, and the 6-month OS rate (95% CI) was 82.7% (67–91.3; Supplementary Fig. S3).

#### Investigator assessed antitumor activity by subgroups

The cORRs were generally consistent across prespecified subgroups, with some notable differences (Supplementary Fig. S4). The cORR for White (*n* = 29) and non-White (*n* = 11) patients was 41.4% and 36.4%, respectively. Patients who had received prior radiotherapy (*n* = 23) had a cORR of 47.8%, whereas patients who had not received radiotherapy (*n* = 18) had a cORR of 27.8%.

The cORR for patients without prior lines of systemic therapy used as part of multimodal curative-intent therapy (*n* = 27) and those with at least one line of prior therapy (*n* = 14) was 29.6% and 57.1%, respectively. Patients who had received prior platinum-based treatment as part of multimodal curative-intent therapy (*n* = 14) had a cORR of 57.1%, whereas those who had not previously received platinum-based treatment (*n* = 27) had a cORR of 29.6%.

In patients with PD-L1 CPS 1 to 19 (*n* = 16), the cORR was 43.8%, and in patients with PD-L1 CPS ≥20 (*n* = 25), the cORR was 36%. In patients with p16-positive oropharyngeal cancer (*n* = 11), the cORR was 81.8%, in contrast to 23.3% in patients with non-HPV–associated disease (*n* = 30). In an exploratory analysis, median nectin-4 H scores were similar between responders and nonresponders. Although higher nectin-4 H scores were seen in patients with a best response of CR (*n* = 4), results should be interpreted with caution due to the small sample size (Supplementary Fig. S5).

#### Antitumor activity per BICR

Twelve patients achieved a response (all PR) per independent central review, with a cORR (95% CI) of 29.3% (16.1–45.5; Supplementary Table S2). The median PFS (95% CI) per independent central review was 5.3 (2.3–NE) months, with a 6-month PFS rate (95% CI) of 47.7% (31–62.6) and was consistent with investigator assessments.

### Safety

Thirty-eight patients (92.7%) experienced TRAEs, and nine patients (22%) had serious TRAEs (Table 3). Grade ≥3 TRAEs were experienced by 41.5% of patients, of which the most common were maculopapular rash (7.3%), acute kidney injury (4.9%), fatigue (4.9%), decreased lymphocyte count (4.9%), and decreased neutrophil count (4.9%; Table 4). TRAEs led to EV dose reductions in 22% of patients (Supplementary Table S3). TRAEs led to EV dose interruptions in 46.3% of patients and pembrolizumab dose interruptions in 46.3% of patients. TRAEs led to the discontinuation of EV in 9.8% of patients and the discontinuation of pembrolizumab in 12.2% of patients.

**Table 3.** Overview of TEAEs, TRAEs, and death.

Parameter, *n* (%)	R/M HNSCC*n* = 41	
	Any TEAE	Any TRAE^a^
Number of patients with AEs	41 (100)	38 (92.7)
Grade ≥3 AEs	29 (70.7)	17 (41.5)
Serious AEs	21 (51.2)	9 (22)
AE leading to the withdrawal of any study drug	7 (17.1)	6 (14.6)
AE leading to the withdrawal of EV	5 (12.2)	4 (9.8)
AE leading to the withdrawal of pembrolizumab	6 (14.6)	5 (12.2)
AE leading to dose reduction of EV	10 (24.4)	9 (22)
AE leading to dose interruption of any study drug	28 (68.3)	23 (56.1)
AE leading to dose interruption of EV	25 (61)	19 (46.3)
AE leading to dose interruption of pembrolizumab	27 (65.9)	19 (46.3)
AE leading to death	4 (9.8)	1 (2.4)
AE leading to death, excluding disease progression	3 (7.3)	1 (2.4)

Abbreviation: TEAE, treatment-emergent AE.

a Defined as a reasonable possibility that the event may have been caused by EV or pembrolizumab, as assessed by the investigator.

**Table 4.** Most common^a^ any grade and grade ≥3 TRAEs.

TRAEs, *n* (%)	R/M HNSCC *n* = 41	
	Any grade	Grade ≥3
Fatigue	18 (43.9)	2 (4.9)
Pruritus	16 (39)	0
Alopecia	12 (29.3)	0
Nausea	10 (24.4)	1 (2.4)
Maculo-papular rash	10 (24.4)	3 (7.3)
Diarrhea	9 (22)	1 (2.4)
Rash	9 (22)	1 (2.4)
Increased aspartate aminotransferase	8 (19.5)	1 (2.4)
Decreased appetite	8 (19.5)	0
Peripheral sensory neuropathy	8 (19.5)	1 (2.4)
Increased alanine aminotransferase	7 (17.1)	1 (2.4)
Dry skin	7 (17.1)	0
Anemia	6 (14.6)	1 (2.4)
Dysgeusia	6 (14.6)	0
Hypothyroidism	4 (9.8)	0
Decreased lymphocyte count	4 (9.8)	2 (4.9)
Arthralgia	3 (7.3)	0
Dry eye	3 (7.3)	0
Pyrexia	3 (7.3)	0
Blurred vision	3 (7.3)	0
Acute kidney injury	2 (4.9)	2 (4.9)
Increased blood lactate dehydrogenase	2 (4.9)	0
Dry mouth	2 (4.9)	0
Eczema	2 (4.9)	0
Embolism	2 (4.9)	0
Headache	2 (4.9)	0
Peripheral neuropathy	2 (4.9)	0
Decreased neutrophil count	2 (4.9)	2 (4.9)
Peripheral motor neuropathy	2 (4.9)	0
Stomatitis	2 (4.9)	0
Decreased white blood cell count	2 (4.9)	0

a TRAEs reported by at least two patients.

The TRAEs considered to be AEs of special interest for EV (but attributable to either treatment agent or both) were skin reactions (61%; grade ≥3, 9.8%), ocular disorders (22%; grade ≥3, 0%), peripheral neuropathy (31.7%; grade ≥3, 2.4%), and hyperglycemia (2.4%; grade ≥3, 2.4%).

The most common AEs that are considered to be AEs of special interest for pembrolizumab but could be attributable to either treatment agent or both were severe skin reactions (14.6%; grade ≥3, 9.8%), hypothyroidism (9.8%; grade ≥3, 0%), infusion reactions (2.4%; grade ≥3, 2.4%), colitis (2.4%; grade ≥3, 0%), pneumonitis (2.4%; grade ≥3, 0%), and adrenal insufficiency (2.4%; grade ≥3, 0%).

Four patients had an AE that led to death, of which one was considered treatment-related. The cause of death for this patient was acute liver failure (possibly immune-mediated) in the setting of preexisting cirrhosis, along with erythema multiforme leading to a cascade of events that ultimately resulted in multiorgan failure, septic shock, and then death. The patient deaths considered not treatment-related were attributed to a death at home within days of a significant clinical response in one patient; acute hypoxic respiratory failure, likely due to aspiration pneumonia, in a patient with a tracheostomy and Dobhoff tube in place; and disease progression in the third patient.

### Immunogenicity

In patients with no antitherapeutic antibodies against EV detected at baseline (*n* = 35), one patient was positive postbaseline. Three patients had antitherapeutic antibodies against EV at baseline, of which one was negative and two were positive postbaseline.

### Pharmacokinetics

The mean (standard deviation) serum concentration of EV was 33.7 (27.5) μg/mL at the end of infusion on cycle 1 day 1 (*n* = 41) and 0.3 (0.2) μg/mL predose on cycle 2 day 1 (*n* = 39; Supplementary Table S4). The mean (standard deviation) plasma concentration of free (unconjugated) MMAE was 0.4 (0.7) ng/mL at the end of infusion on cycle 1 day 1 (*n* = 41) and 0.4 (0.3) ng/mL predose on cycle 2 day 1 (*n* = 39).

### PROs

The baseline completion rate was 95.1% for the global pain assessment measure. The compliance rate remained relatively stable through cycle 9 (at least 80%, reducing to 60% by the end of cycle 10). A consistent trend, suggestive of improvement (reduction in score from baseline), was observed from baseline through to cycle 9, end, and 30-day follow-up (Supplementary Fig. S6A). However, within the study, the minimally important clinical (MIC) difference was not established, nor was the impact of pain medications assessed.

Using the PRO-CTCAE, the study assessed the patient perspective of two side effects: fatigue and diarrhea during treatment. The baseline completion rate was 68.3%, and the compliance rate remained ≥70% through the end of cycle 10. Throughout the study period, the majority of patients reported infrequent diarrhea (never or rarely) across most study visits, with no marked shifts toward higher frequency reporting over the treatment period; this would suggest limited impact of diarrhea over time (Supplementary Fig. S6B).

Fatigue was assessed from a patient perspective using two questions: (i) fatigue severity and (ii) fatigue interference. Across both questions, most patients reported minimal impact, citing “never or rarely” across most study visits. The end-of-treatment score did suggest increased fatigue in terms of severity and interference versus baseline. However, across all assessments, the proportion of patients citing “frequently or almost constantly” was low. Overall, the shift in frequency from “never or rarely” to “occasionally” would indicate a noticeable impact of fatigue severity and interference over time (Supplementary Fig. S6C and S6D).

The baseline completion rate for the FACT-G question 5, “I am bothered by side effects of treatment,” was 58.5%, and the compliance rate remained ≥70% by the end of cycle 10. Most patients reported a manageable side effect profile from cycle 2 through to the end of cycle 10, citing “a little bit” or “somewhat” bothered. At the end of treatment, a high proportion of patients reported being bothered by side effects citing “somewhat” and “quite a bit” bothered (Supplementary Fig. S6E).

The baseline completion rate for the European Organisation for Research and Treatment of Cancer (EORTC) QLQ-H&N43 was 100%. The compliance rates remained high (>85%) through day 1 of cycle 9 but began to decline after the end of cycle 9. Broadly, there was an improvement in the change from baseline EORTC QLQ-H&N43 in the pain in mouth, problems with shoulder, fear of progression, problems opening mouth, and swelling neck scales (Supplementary Fig. S6F). A deterioration compared with baseline was consistently observed at almost all time points in the swallowing, problems with sense, speech, body image, skin problems, weight loss, problems with wound healing, and neurologic problems scales. Compared with baseline, problems with teeth, dry mouth and sticky saliva, social eating, sexuality, coughing, and social contact scales remained consistent. The authors concluded that no single MIC applied across all HN43 scales. Of note, where MIC scores were available, improvement was observed for dry mouth and sticky saliva at cycle 4 day 1 and cycle 9 day 1, speech problems at cycle 9 day 8, coughing at cycle 7 day 1 and cycle 8 day 1 through to cycle 10 day 8, and finally for the scale problems with swelling in the neck at cycle 4 day 8, cycle 7 day 8, cycle 8 day 1, cycle 9 day 1, cycle 9 day 8, and 30-day follow-up. Similarly, with a MIC score available, the following scales observed deterioration: problems with wound healing from cycle 2 day 8 through the 30-day follow-up, coughing at the end of treatment, body image at cycle 8 day 8, and swallowing from cycle 7 day 1 through cycle 9 day 8.

## Discussion

Despite advancements in treatment for R/M HNSCC, including immunotherapy, first-line treatment options in this patient population are still lacking (17), and patient survival rates remain low (5, 6, 18–20). Thus, more effective treatments for patients with R/M HNSCC are still needed.

In this cohort of the EV-202 study, 16 patients achieved a response, exceeding the protocol-defined threshold of 14 patients to declare promising antitumor activity, and it was often durable, with a 6-month DOR rate of 81.7%. Of note, although only 11 patients had p16-positive oropharyngeal cancer, they had a cORR of 81.8%, in contrast to 23.3% in patients with non–HPV-associated disease, which may have driven the cORR observed in the overall population. Broadly, the cORR was consistent across most subgroups, including patients with PD-L1 CPS 1 to 19 and CPS ≥20. However, subgroup data should be interpreted cautiously due to the small sample size of each subgroup. The difference between investigator-assessed and BICR-assessed cORR (39% and 29.3%, respectively) in this study may reflect four patients who had unmeasurable disease at baseline by BICR assessment based on RECIST v1.1.

Although any cross-study comparisons should be interpreted cautiously, in the present study, the cORR was greater than what was observed with pembrolizumab monotherapy (17%) and similar to what was observed with pembrolizumab and chemotherapy (36%) in historical controls (5). The investigator-assessed cORR was also higher than in cohort 5 of the EV-202 study (23.9%; ref. 11) although cohort 5 enrolled patients who were more heavily pretreated than the patients in this cohort. Median PFS was also longer in the present study (5.1 months) compared with what was observed with pembrolizumab monotherapy (2.3 months) and similar to what was observed with pembrolizumab and chemotherapy (4.9 months; ref. 5).

Nectin-4 expression was detected in the majority of patients with available tumor tissue, and levels were similar between responders and nonresponders, consistent with what has been previously reported for EV monotherapy (11). In the current study, the rate of immunogenicity was low, consistent with what has been previously reported (21). The serum and plasma concentrations of EV and MMAE were broadly consistent with those of patients with R/M head and neck cancer who received EV monotherapy (11). Similar to that observed in la/mUC, combination with pembrolizumab did not affect EV pharmacokinetics (15).

The safety profile in this cohort was consistent with the well-established safety and tolerability profile of EV with pembrolizumab in la/mUC (14). In the current study, grade ≥3 TRAEs were reported in 41.5% of patients versus 55.9% of the 440 patients with la/mUC in the EV-302 study (14). The incidence of common TRAEs, both any grade and grade ≥3, as well as TRAEs of special interest for EV (such as peripheral neuropathy), was also similar to what was previously reported (14). In addition, the rates of TRAEs of special interest for EV were similar between the current study and the cohort of patients with R/M head and neck cancer who received EV monotherapy from the EV-202 study (11). Overall, no new safety signals were identified with EV and pembrolizumab combination treatment. These results indicate that EV with pembrolizumab could potentially be a therapeutic option within the current treatment paradigm for patients with R/M HNSCC.

Notably, among the PROs evaluated in this study, patients showed a trend of improvement over time in the global pain scale assessment, and EORTC QLQ-H&N43 results revealed that there was an improvement in five scales, including pain in the mouth. Although the PROs are informative for understanding the impact of symptoms and side effects of treatment for HNSCC, they cannot inform the impact of EV and pembrolizumab versus the current standard of care. Future research is needed on which PROs are meaningful to improve or maintain in patients with R/M HNSCC.

The limitations of this study include its small sample size and lack of a prospective comparator arm, which may affect the interpretation of the efficacy analyses. Two patients discontinued the study due to toxicity or patient decision but continued with pembrolizumab monotherapy as the standard of care outside of the study. As these two patients discontinued the study, pembrolizumab monotherapy exposure data were not captured, and these patients were censored for DOR and PFS analyses at time points before their discontinuation as imaging data after their discontinuation were not collected. The results were compared with historical controls that may differ in patient populations, protocols, and other variables. Patients also did not undergo any testing to confirm their HPV status. This analysis had a short follow-up, which meant survival outcomes were not mature, but patient follow-up is ongoing. Additional research is necessary to fully examine EV with pembrolizumab in this patient population.

Although this study observed some outcome differences by clinical characteristics, in the existing scientific literature, no validated predictive biomarkers have been established for EV with pembrolizumab (12). Nectin-4 has been evaluated as a potential biomarker, but in this study, differences in nectin-4 expression were not robustly associated with response. Neither were PD-L1 CPS of 1 to 19 or ≥20. Although differences in cORR were observed in patients with p16-positive oropharyngeal cancer and patients with non–HPV-associated disease, these results should be interpreted with caution because HPV/p16 was not centrally assessed, and the number of patients included in these subgroups was small. Further evaluation is needed to understand whether a relationship between HPV/p16 positivity and response to EV with pembrolizumab exists in patients with R/M HNSCC.

In conclusion, EV with pembrolizumab demonstrated clinical activity as a first-line treatment in patients with PD-L1 CPS ≥1 R/M HNSCC. Treatment led to antitumor activity, with durable responses noted in some patients, and a safety profile that was consistent with previous reports, reinforcing the manageable safety profile of EV with pembrolizumab. In the future, it may be worthwhile to explore the combination of antibody–drug conjugates targeting nectin-4 and PD-1 inhibitors in R/M HNSCC.

## Supplementary Material

Supplementary Data 1 Supplementary Methods, 4 Supplementary Tables, and 6 Supplementary Figures

## Data Availability

Details for how researchers may request access to anonymized participant-level data, trial-level data, and protocols from Astellas-sponsored clinical trials can be found at https://www.clinicaltrials.astellas.com/transparency/.
