# Supplementary material for: Enfortumab Vedotin plus Pembrolizumab as First-line Treatment in Recurrent or Metastatic Head and Neck Squamous Cell Carcinoma: Results from EV-202 Cohort 9
Source: Clin Cancer Res. 2026 May 5;32(16):3531–9. doi: 10.1158/1078-0432.CCR-25-4650 (PMC13473864; doi:10.1158/1078-0432.CCR-25-4650)
Supplement: Supplementary file 1 [file ccr-25-4650_supplementary_data_1_suppsd_1.docx]

# Supplementary information

## Supplementary Methods

### Exploratory outcomes

For immunogenicity analyses, blood samples for detecting antitherapeutic antibodies were collected within 24 hours prior to enfortumab vedotin (EV) administration on day 1 of cycles 1, 2, 4, 7,12, at the end of treatment (±1 week), and at the 30-day follow-up (±1 week). Analysis of antitherapeutic antibodies in serum was performed using validated methods at bioanalytical laboratories specified by the study sponsor.

For pharmacokinetic analyses, blood samples were collected within 24 hours prior to EV administration on day 1 and at the end of infusion (within 15 minutes) on day 1. Additional samples were collected predose on day 1 of cycle 2, 4, 7, 12, at the end of treatment (±1 week), and at the 30-day follow-up (±1 week). Validated assays were used to measure the concentration of EV and MMAE in serum or plasma. Pharmacokinetic analyses were performed from the pharmacokinetic analysis set which included all patients who received at least 1 dose of EV and pembrolizumab and who had at least 1 blood sample collected and assayed for the measurement of EV and monomethyl auristatin E (MMAE). For these analyses, the mean and standard deviation of EV and MMAE concentrations at selected time points are presented.

Patient-reported outcomes were evaluated with global pain assessment (worst pain in the last 24 hours), European Organisation for Research and Treatment of Cancer Quality of Life Questionnaire - Head and Neck 43 items (EORTC QLQ H&N43), Functional Assessment of Cancer Therapy – General (FACT-G) question GP5 (scoring the statement “I am bothered by side effects of treatment”), and symptom-specific questions (diarrhea and fatigue) from Patient-Reported Outcomes version of the Common Terminology Criteria for Adverse Events (PRO-CTCAE). Patients completed paper questionnaires at baseline, before dosing on day 1 of each cycle, and at the end of treatment. Completion rates at each visit were calculated as the number of patients with at least 1 question completed divided from the total number of patients. Compliance rates at each visit were calculated as the number of patients with at least 1 question completed divided by the number of patients who are expected to have a PRO assessment. This study did not derive values to inform a change considered as minimally important clinical difference across all EORTC QLQ HN43 scales; therefore, to interpret a change in score from baseline, we considered the findings of Singer et al. 2024 (1) that reported these values from 503 patients across 15 countries undergoing treatment for head and neck cancer.

## Supplementary Tables

**Supplementary Table S1:** Representativeness of Study Participants

| **Cancer type(s)/subtype(s)/stage(s)/condition** | Recurrent or metastatic HNSCC |
| --- | --- |
| Other considerations | In patients with HNSCC, approximately 25% to 50% develop recurrent or metastatic disease after primary treatment (2,3).  The data below refers to statistics for lip, oral cavity, salivary gland, pharynx, and larynx cancers combined (4) or oral cavity and pharynx cancers (5). |
| Considerations related to: | |
| Sex | In 2022, the global age-standardized incidence of HNC in males was 15.3 per 100,000 persons and 4.6 per 100,000 persons in females (4). |
| Age | In 2022, the global age-standardized incidence of HNC in older (50–74 years) patients was 36.1 per 100,000 persons and 4.6 per 100,000 persons in younger (15–49 years) patients (4). |
| Race/ethnicity | From 2018–2022, in the United States, the age-standardized incidence of oral cavity and pharynx cancer was broadly similar between Hispanic, Black, and Pacific Islanders (10.3–12.9 per 100,000 persons in males and 4.7–5.8 per 100,000 persons in females). Whites and American Indian/Alaska Natives had greater age-standardized incidences of 17.7–20.6 per 100,000 and 6.4–7.4 per 100,000 in males and females, respectively (5). |
| Geography | In 2022, the global age-standardized incidence of HNC in North America was 10.7 per 100,000 persons,6.4–16.2 per 100,000 persons in Eastern, South-Eastern, and South-Central Asia, and 9.8–12.9 per 100,000 persons in Europe (4). |
| Overall representativeness of this study | The median age of participants in our study (67.0 years) was similar the median age of oral cavity and pharynx cancer diagnosis of 65 years in the United States (5).  The incidence of HNC is greater in males compared with females, which was reflected in this study with most (75.6%) patients being male.  In our study, most patients were from North America and were White ethnicity, which reflects the regional trends observed in the global incidences of HNC (4). |

HNC, head and neck cancer; HNSCC, head and neck squamous cell carcinoma.

**Supplementary Table S2:** Summary of Response by Independent Central Review

|  | **R/M HNSCC (n=41)** |
| --- | --- |
| Best confirmed overall response, n (%)^a^ |  |
| Confirmed complete response | 0 |
| Confirmed partial response | 12 (29.3) |
| Stable disease | 14 (34.1) |
| Progressive disease | 13 (31.7) |
| Not evaluable | 2 (4.9) |
| Confirmed objective response rate, n (%)^b^ | 12 (29.3) |
| 95% CI^c^ | 16.1–45.5 |
| Disease control rate, n (%)^d^ | 26 (63.4) |
| 95% CI^c^ | 46.9–77.9 |

^a^Definition of best overall response followed RECIST v1.1. Complete and partial responses must have been confirmed by 2 scans, a minimum of 4 weeks apart. The minimum duration for stable disease was 49 days after the date of the first dose of EV or pembrolizumab.

^b^Confirmed objective response rate was defined as the proportion of patients whose best overall response was a confirmed complete response or a partial response as per RECIST v1.1.

^c^Calculated using the Clopper-Pearson method.

^d^Disease control rate was defined as the proportion of patients who have a best overall response of confirmed complete response, partial response, or stable disease (≥7 weeks).

CI, confidence intervals; EV, enfortumab vedotin; HNSCC, head and neck squamous cell carcinoma; RECIST v1.1, Response Evaluation Criteria in Solid Tumors version 1.1; R/M, recurrent or metastatic.

**Supplementary Table S3:** Treatment-Related Adverse Events That led to Dose Reductions, Interruptions, and Withdrawal of Treatment

| **Adverse event, n (%)** | **R/M HNSCC (n=41)** | |
| --- | --- | --- |
| Dose reductions of EV | 9 (22.0) | |
| Maculopapular rash | 5 (12.2) | |
| Peripheral sensory neuropathy | 2 (4.9) | |
| Diarrhea | 1 (2.4) | |
| Fatigue | 1 (2.4) | |
| Hyperglycemia | 1 (2.4) | |
| Interstitial lung disease | 1 (2.4) | |
| Dermatitis bullous | 1 (2.4) | |
| Eczema | 1 (2.4) | |
|  | **EV (n=41)** | **Pembrolizumab (n=41)** |
| Dose interruption | 19 (46.3) | 19 (46.3) |
| Peripheral sensory neuropathy | 3 (7.3) | 2 (4.9) |
| Nausea | 2 (4.9) | 2 (4.9) |
| Fatigue | 2 (4.9) | 3 (7.3) |
| Increased alanine aminotransferase | 2 (4.9) | 1 (2.4) |
| Decreased neutrophil count | 2 (4.9) | 2 (4.9) |
| Decreased appetite | 2 (4.9) | 2 (4.9) |
| Peripheral motor neuropathy | 2 (4.9) | 1 (2.4) |
| Maculopapular rash | 2 (4.9) | 4 (9.8) |
| Diarrhea | 1 (2.4) | 1 (2.4) |
| Stomatitis | 1 (2.4) | 0 |
| Gait disturbance | 1 (2.4) | 0 |
| Hypertransaminasemia | 1 (2.4) | 1 (2.4) |
| Conjunctivitis | 1 (2.4) | 0 |
| Enterocolitis infectious | 1 (2.4) | 1 (2.4) |
| Increased blood alkaline phosphatase | 1 (2.4) | 1 (2.4) |
| Increased blood creatinine | 1 (2.4) | 0 |
| Decreased white blood cell count | 1 (2.4) | 1 (2.4) |
| Arthralgia | 1 (2.4) | 1 (2.4) |
| Muscular weakness | 1 (2.4) | 0 |
| Dizziness | 1 (2.4) | 1 (2.4) |
| Acute kidney injury | 1 (2.4) | 0 |
| Interstitial lung disease | 1 (2.4) | 1 (2.4) |
| Dermatitis bullous | 1 (2.4) | 0 |
| Erythema multiforme | 1 (2.4) | 1 (2.4) |
| Rash | 1 (2.4) | 1 (2.4) |
| Embolism | 1 (2.4) | 1 (2.4) |
| Hyperglycemia | 0 | 1 (2.4) |
| Eczema | 0 | 1 (2.4) |
|  | **EV (n=41)** | **Pembrolizumab (n=41)** |
| Withdrawal of treatment | 4 (9.8) | 5 (12.2) |
| Increased alanine aminotransferase | 1 (2.4) | 1 (2.4) |
| Increased aspartate aminotransferase | 1 (2.4) | 1 (2.4) |
| Multiple organ dysfunction syndrome | 1 (2.4) | 1 (2.4) |
| Peripheral sensory neuropathy | 1 (2.4) | 1 (2.4) |
| Maculopapular rash | 1 (2.4) | 0 |
| Acute kidney injury | 0 | 1 (2.4) |
| Increased blood creatinine | 0 | 1 (2.4) |
| Hypertransaminasemia | 0 | 1 (2.4) |

EV, enfortumab vedotin; HNSCC, head and neck squamous cell carcinoma; R/M, recurrent or metastatic.

**Supplementary Table S4:** Serum Concentrations of EV and Plasma Concentrations of Free MMAE

| **EV serum concentrations, µg/mL** | | | | | | | | | |
| --- | --- | --- | --- | --- | --- | --- | --- | --- | --- |
|  | **C1D1** | | **C1D8** | **C2D1** | **C4D1** | **C7D1** | **C12D1** | **EOT** | **Follow-up** |
|  | **Predose** | **EOI** | **Predose** | **Predose** | **Predose** | **Predose** | **Predose** |  |  |
| n | 41 | 41 | 39 | 39 | 32 | 18 | 6 | 17 | 10 |
| Mean  (SD) | 0.007 (N/A) | 33.7  (27.5) | 0.887  (0.271) | 0.331  (0.234) | 0.490  (0.371) | 0.533 (0.541) | 0.302  (0.322) | 0.325  (0.244) | 0.024  (N/A) |
| %CV | N/A | 81.6 | 30.6 | 70.7 | 75.8 | 101 | 106 | 75.2 | N/A |
| Median (range) | 0  (0–0.178) | 28.0  (19.6–195) | 0.917  (0.267–1.41) | 0.292  (0–1.26) | 0.459  (0–1.64) | 0.343  (0–2.20) | 0.206  (0–0.711) | 0.323  (0–0.745) | 0  (0–0.151) |
| GM | N/A | 29.8 | 0.8 | N/A | N/A | N/A | N/A | N/A | N/A |
| Geo %CV | N/A | 0.4 | 0.4 | N/A | N/A | N/A | N/A | N/A | N/A |
| **MMAE plasma concentrations, µg/mL** | | | | | | | | | |
|  | **C1D1** | | **C1D8** | **C2D1** | **C4D1** | **C7D1** | **C12D1** | **EOT** | **Follow-up** |
|  | **Predose** | **EOI** | **Predose** | **Predose** | **Predose** | **Predose** | **Predose** |  |  |
| n | 41 | 41 | 39 | 39 | 32 | 18 | 7 | 17 | 11 |
| Mean  (SD) | 0  (N/A) | 0.413  (0.678) | 1.43 (0.913) | 0.359  (0.250) | 0.381 (0.324) | 0.254 (0.254) | 0.250 (0.386) | 0.376 (0.522) | 0.016 (0.018) |
| %CV | NA | 164 | 64.0 | 69.6 | 85.0 | 100 | 154 | 139 | 112 |
| Median  (range) | 0 (0) | 0.149  (0.027–3.22) | 1.08  (0.507–4.51) | 0.295 (0–0.944) | 0.291  (0–1.37) | 0.177  (0–0.986) | 0.035  (0–1.02) | 0.238  (0–2.18) | 0.015 (0–0.055) |
| GM | N/A | 0.19 | 1.2 | N/A | N/A | N/A | N/A | N/A | N/A |
| Geo %CV | N/A | 1.7 | 0.6 | N/A | N/A | N/A | N/A | N/A | N/A |

Concentrations below the lower limit of quantification (0.01 ng/mL) are set to zero. GM and Geo %CV were not calculated if at least 1 concentration or parameter value was equal to 0. If more than 50% of concentrations are below the lower limit of quantification at a given time point, SD and %CV were calculated.

CV, coefficient of variation; EOI, end of infusion; EOT, end of treatment; EV, enfortumab vedotin; Geo, geometric; GM, geometric mean; MMAE, monomethyl auristatin E; N/A, not applicable; SD, standard deviation.

## Supplementary Figures

**Supplementary Figure S1:** Cohort 9 Study Schema

^a^Local PD-L1 test results may be used for eligibility and should be submitted to confirm positive PD-L1 status (CPS ≥1). If local testing was unavailable, then archival or fresh tissue must have been submitted for central testing prior to enrollment; central PD-L1 testing results were required prior to patient enrollment.

^b^Disease assessment will be performed at screening/baseline. The first on-study disease assessment will be 9 weeks (± 7 days) from the first dose of the study treatment and then every 6 weeks (± 7 days) throughout the study until the patient has radiologically-confirmed disease progression, initiates a new subsequent anticancer therapy, dies, withdraws consent, is lost to follow-up, or the study closes, whichever comes first. A confirmatory imaging scan is required 4 weeks (28 days + 7-day window) after the first response. After 18 months on study treatment, the frequency of response assessments will be reduced to every 9 weeks (84 days ± 7 days).
1L, first-line; CPS, combined positive score; HNSCC, head and neck squamous cell carcinoma; PD-L1, programmed death-ligand 1; R/M, recurrent or metastatic.

**Supplementary Figure S2:** Kaplan-Meier Plot of Duration of Response per Investigator Assessment

CI, confidence interval; NE, not estimable.

**Supplementary Figure S3:** Overall Survival

CI, confidence interval; NE, not estimable.

Supplementary Figure S4: Subgroup Analysis of Confirmed Objective Response Rate per Investigator Assessment

The blue solid line and grey dashed lines represent the cORR (95% CI) of the overall population.

^a^CIs were estimated using the Clopper-Pearson method.

^b^Prior therapy for HNSCC with surgery and/or radiation, with no evidence at the time of enrollment of distant metastatic disease.

^c^Includes prior systemic therapy in neoadjuvant, induction, concurrent with radiotherapy, and/or adjuvant settings.

^d^Responder includes patients who had CR or PR as their best response. Nonresponder includes patients who had stable disease or progressive disease as their best response.

^e^Based on local laboratory PD-L1 test results.

^f^For oropharyngeal squamous cell carcinoma only, patients without oropharynx cancer were considered HPV negative.

BMI, body mass index; CI, confidence interval; CPS, combined positive score; CR, complete response; ECOG PS, Eastern Cooperative Oncology Group performance status; HNSCC, head and neck squamous cell carcinoma; HPV, human papilloma virus; ORR, objective response rate; PD-L1, programmed death-ligand 1; PR, partial response.

**Supplementary Figure S5:** Baseline Nectin-4 H Score by RECIST v1.1 Best Overall Response in Response Groups (A) and by Individual Response (B), per Investigator Assessment

BOR, best overall response; CR, complete response; Max, maximum; Min, minimum; NE, not estimable; PD, progressive disease; PR, partial response; RECIST v1.1, Response Evaluation Criteria in Solid Tumors version 1.1; SD, stable disease; Std, standard deviation.

**Supplementary Figure S6:** (A) Global Pain Assessment, (B) PRO-CTCAE – Diarrhea, (C) PRO-CTCAE – Fatigue Severity, (D) PRO-CTCAE – Fatigue Interference, (E) FACT-G Question 05, (F) EORTC QlQ-H&N43 Summary

^a^Clinically meaningful improvement or deterioration data based on prior literature is not available.
C, cycle; D, day; EORTC QLQ-H&N43, European Organisation for Research and Treatment of Cancer Quality of Life Questionnaire – Head and Neck Cancer Module; FACT-G, Functional Assessment of Cancer Therapy – General; FU, follow-up; MIC, minimally important change; PRO, patient-reported outcome; PRO-CTCAE, Patient-Reported Outcomes version of the Common Terminology Criteria for Adverse Events; trt, treatment.

## Supplementary References

1. Singer S, Hammerlid E, Tomaszewska IM, Amdal CD, Herlofson BB, Santos M, et al: The European Organisation for Research and Treatment of Cancer head and neck cancer module (EORTC QLQ-HN43): estimates for minimal important difference and minimal important change. *Eur J Cancer* **2024**;212:115062.
2. Leeman JE, Li J-G, Pei X, Venigalla P, Zumsteg ZS, Katsoulakis E, et al. Patterns of treatment failure and postrecurrence outcomes among patients with locally advanced head and neck squamous cell carcinoma after chemoradiotherapy using modern radiation techniques. *JAMA Oncology* **2017**;3:1487–94.
3. Argiris A, Karamouzis MV, Raben D, Ferris RL. Head and neck cancer. *Lancet* **2008**;371:1695–709.
4. Huang J, MSocSc SHC, MSocSc MC, Chan SC, Yuan J, Zhang L, et al. Incidence, risk factors, and epidemiological trends of head and neck cancer: a global analysis. *MedComm (2020)*. **2025**;6:e70467.
5. National Cancer Institute, Surveillance, Epidemiology, and End Results Program (SEER). Cancer Stat Facts: Oral Cavity and Pharynx Cancer. Available at: https://seer.cancer.gov/statfacts/html/oralcav.html. [cited December 05, 2025].
